# Splicing Factor 3a Subunit 1 Promotes Colorectal Cancer Growth via Anti-Apoptotic Effects of Syntaxin12

**DOI:** 10.3390/ijms27031195

**Published:** 2026-01-24

**Authors:** Takahiro Sasaki, Hiroaki Konishi, Tatsuya Dokoshi, Aki Sakatani, Hiroki Tanaka, Koji Yamamoto, Keitaro Takahashi, Katsuyoshi Ando, Nobuhiro Ueno, Shin Kashima, Kentaro Moriichi, Hiroki Tanabe, Toshikatsu Okumura, Mikihiro Fujiya

**Affiliations:** 1 Division of Gastroenterology, Department of Internal Medicine, Asahikawa Medical University, Midorigaoka-Higashi 2-1-1-1, Asahikawa 078-8510, Hokkaido, Japan; taka-sas@asahikawa-med.ac.jp (T.S.); ktakaha@asahikawa-med.ac.jp (K.T.); k-ando@asahikawa-med.ac.jp (K.A.); u-eno@asahikawa-med.ac.jp (N.U.); shin1014@asahikawa-med.ac.jp (S.K.); morimori@asahikawa-med.ac.jp (K.M.); tant@asahikawa-med.ac.jp (H.T.); okumurat@asahikawa-med.ac.jp (T.O.); fjym@asahikawa-med.ac.jp (M.F.); 2 Department of Gastroenterology and Advanced Medical Sciences, Asahikawa Medical University, Midorigaoka-Higashi 2-1-1-1, Asahikawa 078-8510, Hokkaido, Japan; kyama.kobe@gmail.com; 3 Department of Gastroenterological Sciences, Asahikawa Medical University, Midorigaoka-Higashi 2-1-1-1, Asahikawa 078-8510, Hokkaido, Japan; ta1983@asahikawa-med.ac.jp (T.D.); sakatani@asahikawa-med.ac.jp (A.S.); 4 Division of Tumor Pathology, Department of Pathology, Asahikawa Medical University, Midorigaoka-Higashi 2-1-1-1, Asahikawa 078-8510, Hokkaido, Japan; hiroki-t@asahikawa-med.ac.jp

**Keywords:** RNA binding protein, colorectal cancer, splicing factor 3A1, Syntaxin 12, apoptosis

## Abstract

RNA dysregulation mediated by aberrant RNA-binding proteins (RBPs) is closely associated with tumorigenesis. However, the tumorigenic mechanisms of each RBP remained unclear. In this study, we demonstrate that downregulation of Splicing factor 3A1 (SF3A1) markedly suppressed the proliferation of colorectal cancer (CRC) cells, with minimal cytotoxicity observed in non-cancerous epithelial cells. The tumor-promoting function of SF3A1 was further validated in an HCT116 xenograft mouse model. Multiple apoptosis assays—including TdT-mediated dUTP nick end labeling (TUNEL) staining, poly-ADP-ribose polymerase (PARP) immunoblotting, and caspase-3/7 activity measurements—showed that SF3A1 inhibited apoptotic signaling in CRC cells. Transcriptome analysis, combined with RNA-immunoprecipitation (RIP), identified Syntaxin 12 (STX12) as a downstream effector of SF3A1. Knockdown of STX12 induced apoptosis in CRC cells but had no effect on the viability of non-cancerous HCEC-1CT epithelial cells. Furthermore, STX12 mRNA levels were significantly reduced following SF3A1 knockdown, indicating that SF3A1-mediated stabilization of STX12 contributes to apoptosis resistance in CRC cells. Collectively, our findings establish that SF3A1 promotes CRC progression by stabilizing STX12 mRNA and selectively inhibiting apoptosis in malignant cells, thereby identifying the SF3A1–STX12 regulatory axis as a novel and selective therapeutic target for CRC.

## 1. Introduction

The treatment landscape for colorectal cancer (CRC) has significantly advanced with the development of molecularly targeted therapies, immune checkpoint inhibitors, and combination chemotherapy employing classical cytotoxic agents such as 5-fluorouracil (5-FU) [1,2]. Despite these advancements, mortality in patients with advanced CRC remains high, highlighting the urgent need for novel therapeutic targets [3].

RNA-binding proteins (RBPs) have emerged as critical regulators of tumorigenesis through diverse mechanisms, including the stabilization of mRNAs and non-coding RNAs, as well as the regulation of cancer-specific alternative splicing [4,5]. Dysregulation of RBPs can selectively reprogram cancer cell survival pathways without necessarily altering global gene expression levels, highlighting their potential as attractive therapeutic targets. Indeed, several RBPs are aberrantly expressed in CRC and have been implicated in tumor growth, metabolic reprogramming, and resistance to apoptosis [6,7,8,9,10,11].

RBPs also contribute to tumorigenesis through post-translational modifications (PTM), such as phosphorylation [12,13,14,15]. To systematically identify RBPs with tumor-promoting functions, we conducted a high-throughput siRNA screen targeting 416 human RBPs across various gastrointestinal cancer cell lines, including colorectal, esophageal, and pancreatic cancer cells. This functional screening, combined with cDNA array expression profiling, identified 12 candidate RBPs—including splicing factor 3A1 (SF3A1)—as potential regulators of cancer progression, potentially through post-translational mechanisms [16].

SF3A1 is a subunit of the SF3a complex, an integral component of U2 small nuclear ribonucleoprotein (snRNP), which is essential for intron recognition and pre-mRNA splicing [17]. Our previous investigation suggested that SF3A1 promotes cancer cell progression without marked differences in expression between tumor and normal cells [16]. Furthermore, genetic alterations of SF3A1 have been implicated in pancreatic cancer [18], and SF3A1 polymorphisms have been associated with CRC risk [19]. However, the downstream mRNA targets of SF3A1 in CRC cells and their potential roles in tumor progression remain poorly understood.

In this study, we aimed to elucidate the mechanisms of SF3A1-mediated tumor progression in CRC cells. Through integrated analyses combining RNA immunoprecipitation (RIP) with transcriptome profiling, we identified a set of SF3A1-binding mRNAs, including Syntaxin 12 (STX12). Functional analyses revealed that disruption of the SF3A1–STX12 axis selectively induces apoptosis in CRC cells, providing mechanistic insight into how SF3A1 contributes to tumor cell survival and highlighting RBPs as potential therapeutic targets in CRC.

## 2. Results

### 2.1. SF3A1 Enhances the Tumor Progression in CRC Cells, but Not in Non-Cancerous Cells

**Figure 1:**
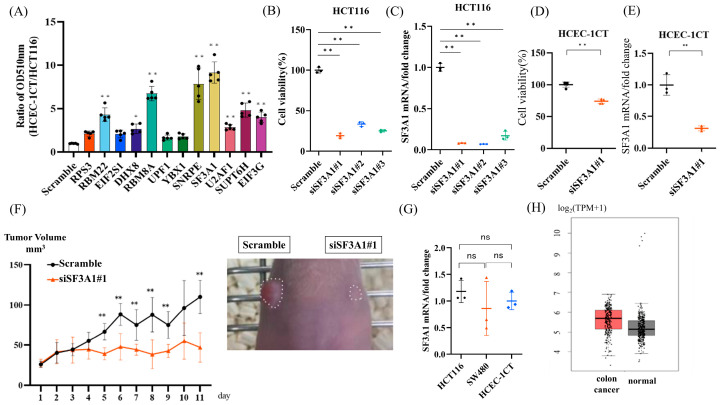
SF3A1 knockdown selectively reduces viability in CRC cells with less impact on non-cancerous cells. (**A**) SRB assays comparing the OD_510_ ratio (HCEC-1CT/HCT116) after siRNA-mediated knockdown of 12 RBPs (n = 5 each) identified SF3A1 as the most CRC-selective candidate. Statistical analyses were performed using one-way analysis of variance (ANOVA), followed by Dunnett’s test. * *p* < 0.05. (**B**) Three distinct siRNAs targeting SF3A1 consistently decreased viability in HCT116 cells (n = 3 each), ruling out off-target effects. Statistical analyses were performed using ANOVA, followed by Dunnett’s test. ** *p* < 0.01. (**C**) Knockdown efficiencies of the three distinct SF3A1 siRNAs were verified by RT-PCR. Statistical analyses were performed using ANOVA, followed by Dunnett’s test. ** *p* < 0.01. (**D**) SF3A1 knockdown exerted only a modest effect on HCEC-1CT cell viability (n = 5). Statistical analyses were performed using Student’s *t*-test. ** *p* < 0.01. (**E**) The knockdown efficacy of SF3A1 in HCEC-1CT. Statistical analyses were performed using Student’s *t*-test. ** *p* < 0.01. (**F**) In vivo, local intratumoral administration of SF3A1 siRNA significantly suppressed the growth of HCT116 xenografts compared with scramble control (scramble: n = 5; siSF3A1: n = 5). Statistical analyses were performed using Student’s *t*-test at each time point. (**G**) Basal SF3A1 mRNA expression levels were comparable among HCT116, SW480, and HCEC-1CT cells (n = 3 each). Statistical analyses were performed using one-way ANOVA, followed by Tukey’s test. ns: not significant. (**H**) GEPIA revealed no significant differences in SF3A1 expression between CRC tissues (n = 275) and normal tissues (n = 349). * *p* < 0.05, ** *p* < 0.01. Error bars represent mean ± SD.

Our previous study identified 12 RBPs—RPS3, RBM22, EIF2S1, DHX8, RBM8A, UPF1, YBX1, SNRPE, SF3A1, U2AF1, SUPT6H, and EIF3G—as exhibiting tumor-promoting properties without any expressional/mutational changes between cancer and non-cancerous cells [16]. To evaluate their functional roles in CRC cells, we performed sulforhodamine B (SRB) assays following siRNA-mediated knockdown of each RBP in the CRC cell line HCT116 and the non-cancerous colon epithelial cell line HCEC-1CT. The ratio of optical density (OD) 510 nm (HCEC-1CT/HCT116) indicated that, among the 12 candidates, SF3A1 knockdown exhibited a significant cytotoxic effect on HCT116 cells, whereas it had a minimal impact on HCEC-1CT cells (Figure 1A). To exclude potential off-target effects, we treated HCT116 cells with three different siRNAs targeting SF3A1. All three siRNAs significantly reduced cell viability (Figure 1B), with knockdown efficiency validated by qPCR (Figure 1C). Similar growth-suppressive effect was observed in another colorectal cancer cell line, SW480 (Figure S1A), whereas the effect on HCEC-1CT cells remained limited (Figure 1D) (the knockdown efficacy of each cell line was confirmed by RT-PCR, Figure S1B and Figure 1E). To assess the in vivo relevance of SF3A1, HCT116 cells were implanted subcutaneously on both flanks of nude mice, followed by intratumoral injection of either SF3A1 siRNA or scrambled control RNA. Consistent with the in vitro findings, SF3A1 knockdown markedly inhibited tumor growth (Figure 1F and Figure S2). We next compared the endogenous expression levels of SF3A1 in CRC cells and non-cancerous colon epithelial cells. RT-PCR confirmed that SF3A1 mRNA expression levels were comparable between CRC cells (HCT116 and SW480) and non-cancerous colon epithelial cells (HCEC-1CT) (Figure 1G). Analysis of public datasets using GEPIA (http://gepia.cancer-pku.cn/ 21 December 2020) further supported this finding, showing no significant difference in SF3A1 expression between CRC tumor samples (n = 275) and normal tissues (n = 349) (Figure 1H). Collectively, these results suggest that SF3A1 promotes tumor growth selectively in CRC cells, despite similar expression levels in non-cancerous cells.

### 2.2. SF3A1 Inhibits Apoptosis of CRC Cells

**Figure 2:**
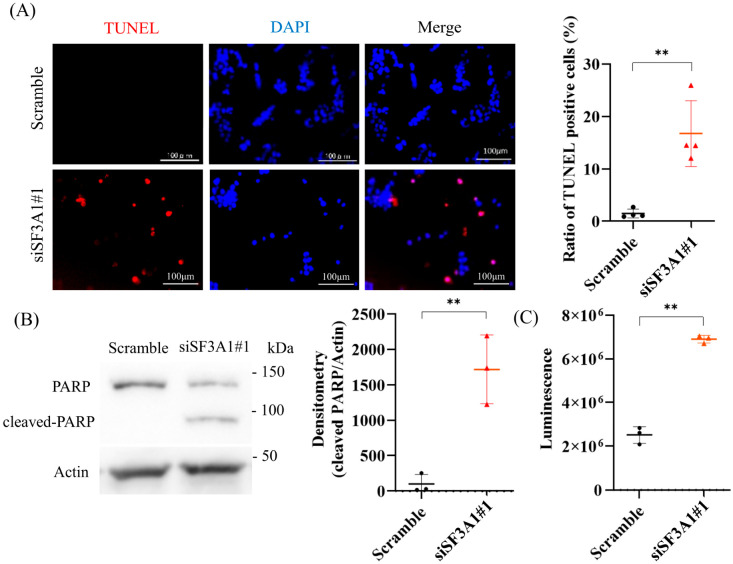
SF3A1 inhibits apoptosis of CRC cells. (**A**) TUNEL staining revealed that SF3A1 knockdown significantly increased the proportion of TUNEL-positive cells in HCT116 cells (n = 4). Statistical analyses were performed using Student’s *t*-test. (**B**) Western blots showed enhanced PARP cleavage in HCT116 cells following SF3A1 knockdown (n = 3). Statistical analyses were performed using Student’s *t*-test. (**C**) Caspase-Glo 3/7 assay revealed elevated caspase activity in HCT116 cells following SF3A1 knockdown (n = 3). Statistical analyses were performed using Student’s *t*-test. ** *p* < 0.01. Error bars represent mean ± SD.

To elucidate whether SF3A1 primarily influences cell proliferation or survival, we performed Ki-67 immunocytochemistry in HCT116 cells following SF3A1 knockdown. The proportion of Ki-67-positive cells remained unchanged (Figure S3), suggesting that SF3A1 does not regulate proliferation. In contrast, TUNEL staining revealed a significant increase in apoptotic cells upon SF3A1 knockdown in HCT116 cells (Figure 2A). A similar increase in TUNEL-positive cells was also observed in SW480 cells following SF3A1 knockdown, indicating that SF3A1-dependent suppression of apoptosis is conserved across CRC cells (Figure S4). Consistently, Western blotting confirmed enhanced PARP cleavage (Figure 2B), and a Caspase-Glo 3/7 assay showed elevated caspase activity (Figure 2C), collectively indicating activation of apoptotic pathways. Notably, none of these apoptotic changes were observed in HCEC-1CT cells (Figure S5). Taken together, these results indicate that SF3A1 promotes CRC cell survival primarily by suppressing apoptosis rather than by regulating proliferative capacity.

### 2.3. SF3A1 Inhibits Apoptosis of CRC Cells by Stabilizing the mRNA of Syntaxin12 (STX12)

To explore the mechanism by which SF3A1 induces apoptosis in CRC cells, we performed RNA-sequencing analysis in HCT116 cells following SF3A1 knockdown. This analysis identified 476 significantly altered mRNAs, of which 307 were downregulated (Table S1). Gene ontology (GO) analysis of these downregulated mRNAs showed that cell survival pathways, such as nucleotide-excision repair, canonical Wnt signaling pathway, and damaged DNA binding pathway, were significantly suppressed (Table 1), suggesting that suppression of these pathways contributes to apoptosis in SF3A1 downregulated CRC cells.

**Figure 3:**
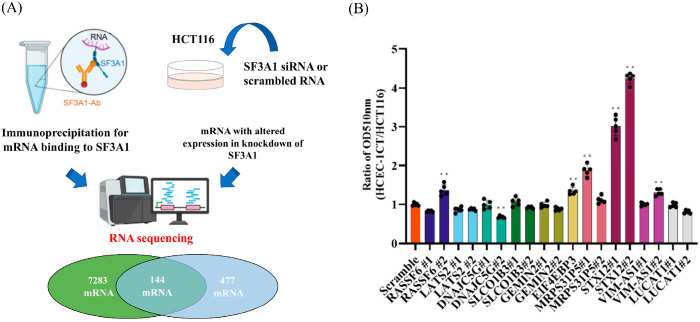
SF3A1 inhibits apoptosis of CRC cells by stabilizing the mRNA of Syntaxin12 (STX12). (**A**) RNA immunoprecipitation (RIP) followed by transcriptome analysis identified 7283 mRNAs that physically interact with SF3A1 in HCT116 cells (fold change > 2, *p* < 0.01, n = 3). RNA-seq analysis of HCT116 cells transfected with SF3A1 siRNA identified 477 mRNAs whose expression was significantly altered upon SF3A1 knockdown (absolute fold change > 2, *p* < 0.01, n = 3). Statistical analyses were performed using Student’s *t*-test. Cross-comparison revealed 144 mRNAs whose expression was both SF3A1-dependent and likely stabilized via direct interaction. (**B**) Two distinct siRNAs targeting each of the top 10 candidate mRNAs were transfected into HCT116 and HCEC-1CT cells, and cell viability was assessed at 72 h using the SRB assay. The ratio of OD_510nm_ (HCEC-1CT/HCT116) was compared (n = 5 each). Among these candidates, STX12 knockdown induced the strongest cytotoxic effect in HCT116 cells while exerting minimal effects on non-cancerous HCEC-1CT cells. Statistical analyses were performed using one-way ANOVA, followed by Dunnett’s test. ** *p* < 0.01. Error bars represent mean ± SD.

Next, we aimed to identify mRNAs that are both directly bound to SF3A1 and downregulated upon its knockdown. We conducted RNA immunoprecipitation (RIP) using an SF3A1-specific antibody in HCT116 cell lysates, with immunoprecipitation efficiency shown in Figure S6. After protein and DNA removal, RNA was extracted from the immunoprecipitated complexes and subjected to transcriptome analysis, which identified 7283 mRNAs that interact with SF3A1 (Table S2). Integrative analysis of RIP-seq and RNA-seq datasets identified 144 mRNAs that were both physically associated with SF3A1 and significantly downregulated upon its silencing, indicating that SF3A1 contributes to their mRNA stability (Figure 3A). From this subset, we selected the top 10 mRNAs showing the most significant downregulation for further investigation (Table 2).

To determine their relevance in CRC, we transfected two distinct siRNAs targeting each of the 10 candidate genes into HCT116 and HCEC-1CT cells and assessed their effects on cell viability. Among them, the knockdown of Syntaxin12 (STX12) showed a maximal cytotoxic effect on HCT116 cells while having minimal impact on HCEC-1CT cells (Figure 3B). Efficient knockdown of STX12 was confirmed by RT-PCR (Figure S7A) and at the protein level by Western blotting (Figure S7B).

**Figure 4:**
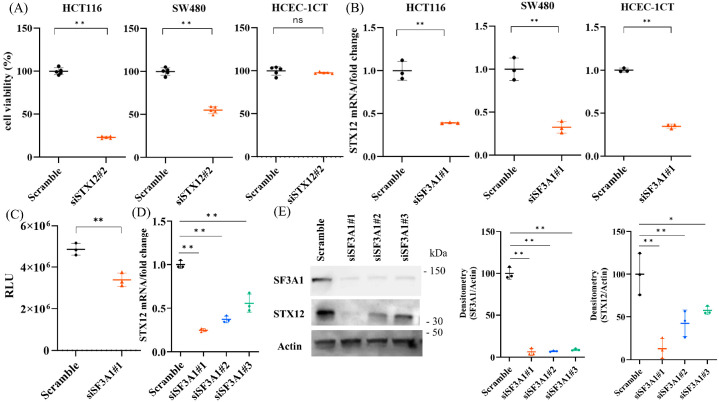
SF3A1 stabilizes STX12 mRNA, promoting tumor progression in CRC cells but not in non-cancerous cells. (**A**) An SRB assay showed that STX12 knockdown led to the growth suppression in cancer cells (HCT116, SW480), but not non-cancerous epithelial cells (HCEC-1CT) (n = 5). Statistical analyses were performed using Student’s *t*-test. ns: not significant. (**B**) RT-PCR confirmed the knockdown efficacy of STX12 in HCT116, SW480, and HCEC-1CT cells following siRNA transfection (n = 3). Statistical analyses were performed using Student’s *t*-test. (**C**) Luciferase reporter assay demonstrated reduced luminescence from the STX12 mRNA construct, but not from the empty vector, in HCT116 cells after SF3A1 knockdown, indicating reduced mRNA stability (n = 3). Statistical analyses were performed using Student’s *t*-test. (**D**) Three independent siRNAs targeting SF3A1 consistently reduced STX12 mRNA (n = 3 each). Statistical analyses were performed using Dunnett’s test. (**E**) Western blots showed that three distinct siRNAs targeting SF3A1 consistently decreased both SF3A1 and STX12 protein expression in HCT116 cells (n = 3 each). Statistical analyses were performed using ANOVA, followed by Dunnett’s test. * *p* < 0.05, ** *p* < 0.01. Error bars represent mean ± SD.

To validate the functional role of STX12 in CRC cell survival, we performed siRNA-mediated knockdown of STX12 in CRC and non-cancerous cell lines. STX12 knockdown significantly reduced the viability of both HCT116 and SW480 colorectal cancer cells, while having minimal effect on HCEC-1CT non-cancerous cells (Figure 4A). Efficient knockdown was confirmed in all cell types (Figure S7C). RT-PCR analysis demonstrated that STX12 mRNA expression was consistently reduced following SF3A1 knockdown in HCT116, SW480, and HCEC-1CT cells (Figure 4B). To further confirm that SF3A1 stabilizes STX12 mRNA, a luciferase reporter assay was conducted in HCT116 cells. The luminescence of the STX12 reporter construct, but not the empty vector, was significantly decreased in HCT116 cells following SF3A1 knockdown (Figure 4C), indicating reduced mRNA stability. Consistently, three distinct SF3A1 siRNAs downregulated STX12 mRNA and protein expression in HCT116 cells (Figure 4D,E). Together, these results suggest that SF3A1 regulates STX12 expression in both cancerous and non-cancerous epithelial cells. STX12 depletion selectively induces apoptosis in CRC cells, suggesting that the SF3A1–STX12 axis plays a cancer-specific role in promoting CRC cell survival.

### 2.4. STX12 Inhibits the Apoptosis in CRC Cells

**Figure 5:**
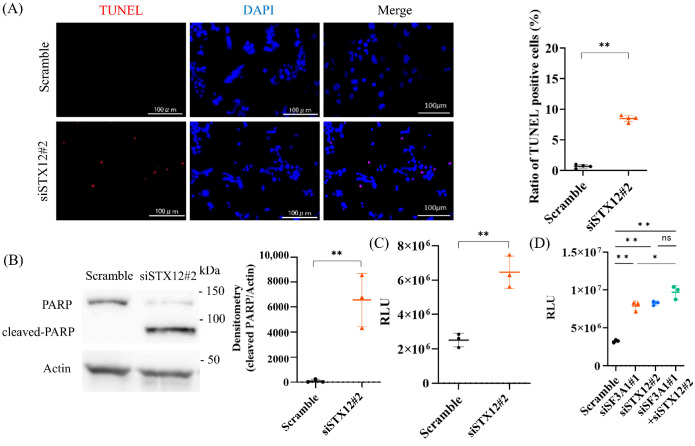
STX12 inhibits apoptosis in CRC cells (**A**) TUNEL staining demonstrated an increase in apoptotic cells in HCT116 cells following STX12 knockdown (n = 4). Statistical analyses were performed using Student’s *t*-test. (**B**) Western blots showed enhanced PARP cleavage in HCT116 cells following STX12 knockdown (n = 3). Statistical analyses were performed using Student’s *t*-test. (**C**) Caspase 3/7 activity was significantly increased in HCT116 cells following STX12 knockdown (n = 3). Statistical analyses were performed using Student’s *t*-test. (**D**) Double knockdown of SF3A1 and STX12 did not further increase caspase-3/7 activity compared with STX12 knockdown alone (n = 3). Statistical analyses were performed using ANOVA followed by Tukey’s post hoc test. ns: not significant. * *p* < 0.05, ** *p* < 0.01. Error bars represent mean ± SD.

Given a recent report showing that STX2 promotes CRC growth [20], we next examined whether STX12 similarly contributes to CRC cell proliferation and survival. TUNEL staining showed a significant increase in apoptotic cells in HCT116 cells following STX12 knockdown, whereas no comparable increase was observed in non-cancerous HCEC-1CT cells (Figure 5A and Figure S9). Consistently, Western blotting demonstrated increased PARP cleavage (Figure 5B), and caspase 3/7 assays revealed elevated caspase activity (Figure 5C). Notably, simultaneous knockdown of SF3A1 and STX12 did not further enhance caspase-3/7 activity compared with STX12 knockdown alone (Figure 5D), suggesting that STX12 acts downstream of SF3A1 in the apoptotic pathway (STX12 mRNA expression shown in Figure S7D). In contrast, immunocytochemistry for Ki-67 showed no significant change in the proportion of proliferating HCT116 cells following STX12 knockdown, indicating that STX12 does not affect cell proliferation (Figure S8). Taken together, these findings identify STX12 as a critical downstream effector of SF3A1-mediated anti-apoptotic signaling that selectively supports survival in CRC cells.

## 3. Discussion

In this study, we demonstrated that the RNA-binding protein SF3A1 promotes CRC progression by suppressing apoptosis through stabilization of STX12 mRNA. Our findings establish the SF3A1–STX12 axis as a cancer-selective survival mechanism that operates in both in vitro and in vivo CRC models.

Our RIP assay and gene reporter assay demonstrated that SF3A1 stabilizes STX12 mRNA through direct binding. Apoptotic assays, including TUNEL staining and caspase-3/7 activity measurements, consistently showed that SF3A1 or STX12 knockdown induces apoptosis in CRC cells, whereas non-cancerous epithelial cells were largely unaffected. These findings indicate that the SF3A1-STX12 axis exerts a cancer-selective anti-apoptotic function rather than broadly regulating cell viability. Furthermore, transcriptomic profiling revealed that SF3A1 knockdown suppressed key survival pathways such as nucleotide excision repair and Wnt signaling, which are critical for the DNA damage response [21] and cellular proliferation [22], respectively. The downregulation of these pathways likely contributes to the enhanced apoptosis observed upon SF3A1 silencing, in addition to the loss of STX12-mediated survival signaling. Collectively, these findings suggest that SF3A1 suppresses cell death signaling in CRC cells by maintaining STX12 expression and preserving survival signaling networks.

Previous investigations have indicated the tumor-promoting potential of STX12 through oncogenic/tumor suppressive signaling pathways such as STAT3 and NF-κB. Lee et al. reported that STX12 expression is upregulated via the ROS/STAT3/NFE2L1 pathway, supporting tumor progression in hepatocellular carcinoma cells [23]. Since STAT3 is a well-established oncogenic driver in CRC [24,25], SF3A1 may promote CRC by stabilizing STAT3-STX12-mediated anti-apoptotic signaling. Additionally, STX12 has been shown to suppress the expression of miR-148a, a microRNA that negatively regulates NF-κB signaling through targeting the 3′-UTR of TLR4 mRNA [26], suggesting an additional STX12-driven survival axis that may operate in CRC. Interestingly, GEPIA showed that the expression of STX12 was not significantly changed in CRC patients (Figure S10), indicating that the oncogenic relevance of STX12 is likely governed by cancer-specific regulatory mechanisms rather than expression levels. Indeed, Malik et al. showed that STX12 is phosphorylated by SGK3 [27], which is known as a downstream effector of phosphatidylinositol 3-kinase (PI3K) oncogenic signaling [28], indicating that PTM, as well as the expression of STX12, plays a crucial role in CRC progression. Such PTM-dependent regulation may explain why STX12 is indispensable in CRC cells but dispensable in non-cancerous epithelial cells. However, the specific apoptotic pathways modulated by STX12 and the contribution of SGK3-dependent phosphorylation remain incompletely understood. Comparative analyses of apoptotic signaling and PTM dynamics in normal versus malignant cells will be essential to elucidate the mechanistic basis of the SF3A1–STX12 axis.

Similarly, SF3A1 expression levels were comparable between CRC and non-cancerous epithelial cells, despite its tumor-specific functional impact. This observation suggests that PTMs of SF3A1 may regulate its RNA-stabilizing activity in cancer cells. This concept parallels our previous findings that phosphorylation of hnRNP A0, an RNA-binding protein, at Ser84, mediated by MAPKAP2, promoted CRC progression by stabilizing oncogenic RAB3GAP1 mRNA [15]. SF3A1 contains 23 serine/threonine residues that may be subject to phosphorylation, potentially contributing to its ability to stabilize mRNA targets such as STX12 (https://www.phosphosite.org/proteinAction.action?id=4183&showAllSites=true, 1 November 2024). Understanding the mechanisms of PTM of SF3A1, including phosphorylation, ubiquitination, glycosylation, acetylation, and others, may lead to the development of novel cancer therapies targeting its tumor-specific functions.

Although SF3A1 knockdown was comparably efficient across CRC cell lines, the inhibitory effect on proliferation was less in SW480 than in HCT116 cells. This suggests variability in SF3A1’s PTM status even among CRC subtypes. Identifying the cancer-specific factors that modulate SF3A1 PTMs may help predict the therapeutic responsiveness of SF3A1-targeted interventions.

Several other RBPs, including RBM22 and U2AF1, have been shown to possess tumor-promoting activities, which may also be regulated through PTMs [29,30]. However, the specific mRNA targets and regulatory mechanisms of these RBPs remain unknown. Our previous investigations have identified various RBPs upregulated in CRC, such as hnRNP A1, hnRNP H1, and RBMXL2, each controlling distinct pathways such as the cell cycle and sphingolipid metabolism [6,7,31,32]. A comprehensive analysis of how each RBP contributes to tumor biology will be essential for designing targeted therapeutic strategies based on RBP expression profiles and modification statuses.

Transcriptome profiling revealed no evidence for activation of alternative RNA-processing mechanisms or upregulation of related RBPs following SF3A1 knockdown. Instead, the dominant transcriptional changes were marked by downregulation of cancer-associated survival pathways, including Wnt signaling, consistent with the impaired cell viability observed following SF3A1 knockdown. While subtle post-transcriptional adaptations cannot be excluded, these findings suggest that compensatory RNA-processing responses are unlikely to be the primary drivers of the observed cellular effects.

Several limitations should be acknowledged. First, it remains unclear whether STX12 mRNA possesses a particular sequence motif that confers preferential binding by SF3A1. Although SF3A1 contains conserved SURP RNA-binding domains, the precise RNA-recognition motif of SF3A1 has not yet been defined. High-resolution approaches such as eCLIP-based crosslinking assays will be required to elucidate SF3A1 RNA-binding specificity. Second, although we validated SF3A1 knockdown effects in SW480 cells, additional CRC subtypes and patient-derived models were not examined, limiting the generalizability of this regulatory axis. Furthermore, the in vivo experiments relied on intratumoral siRNA delivery, which lacks clinical relevance; thus, evaluation using systemic delivery methods and pharmacokinetic characterization will be required to assess translational feasibility.

## 4. Materials and Methods

### 4.1. Ethics

All experiments were performed according to the “Guidelines of the Public Health Service Policy on the Humane Use and Care of Laboratory Animals.” The study received ethical approval for the use of an opt-out methodology from the Medical Ethics Committee of Asahikawa Medical University (Approval No. R5-059: The date of approval 1 April 2023, Approval No. R6-006: The date of approval 1 April 2024). All animal experiments were reported in accordance with the ARRIVE guidelines.

### 4.2. Cell Culture

Human CRC cell lines (HCT116 and SW480) (American Type Culture Collection [ATCC], Manassas, VA, USA) were cultured in McCoy’s 5A medium (HCT116) and Roswell Park Memorial Institute 1640 medium (SW480). The media were supplemented with 10% (vol/vol) fetal bovine serum (FBS) and 50 U/mL penicillin, and cells were maintained at 5% CO_2_ and 37 °C under humidified conditions. Human colon epithelial cells (HCEC-1CT) (Summit Pharmaceuticals International Corporation, Tokyo, Japan) were cultured in ColoUp medium (DMEM/Medium 199 Earle’s, 4 + 1; Biochrom Cat# F0435 and Cat# FG0615, Cambridge, UK). The medium included 4 mM GlutaMAXTM-1 (100×, Gibco, Cat# 35050-038, Waltham, MA, USA), 2% cosmic calf serum (Hyclone, Cat# SH30087, Hyde Park, UT, USA), 20 ng/mL EGF (Sigma Aldrich, Cat# E9644, St. Louis, MO, USA), 10 μg/mL Insulin (Sigma Aldrich, Cat# I9278), 2 μg/mL Apo-Transferrin (Sigma Aldrich, Cat# T2036), 5 nM sodium-selenite (Sigma Aldrich, Cat# S5261), and 1 μg/mL hydrocortisone (Sigma Aldrich, Cat# H0396). The cells were maintained at 5% CO_2_ and 37 °C under humidified conditions.

### 4.3. siRNA and Transfection

All siRNAs used in this study, except for the ones targeting EIF4EBP3, were selected from the Silencer Select siRNA Libraries (Thermo Fisher Scientific, Waltham, MA, USA) (Table S3). EIF4EBP3 siRNA was generated by annealing two synthetic RNAs as follows: sense, 5′-UGGAUUAGAUGUCCAUUUCAA-3′ and antisense, 5′-GAAAUGGACAUCUAAUCCAGU-3′ (Hokkaido System Science Co., Ltd., Sapporo, Japan). A negative control, consisting of a scrambled RNA sequence, was prepared by annealing two synthetic RNAs as follows: sense, 5′-UACGUACUAUCGCGCGGAU-3′ and antisense, 5′-AUCCGCGCGAUAGUACGUA-3′ (Hokkaido System Science Co., Ltd.). Transfection was carried out in triplicate using Lipofectamine RNAiMAX (Thermo Fisher Scientific) following the manufacturer’s instructions for reverse transfection methods.

### 4.4. SRB Assays

The cells were seeded onto 96-well microplates at a density of 0.75 × 10^4^ cells per well. Subsequently, the cells were fixed using a solution of 5% trichloroacetic acid (TCA) at 4 °C for one hour. Following fixation, the plates were rinsed four times with distilled water, dehydrated at room temperature, and then stained with 100 μL/well of 0.057% (wt/vol) SRB powder in 0.1% acetic acid. After staining, the microplates were washed four times with 0.1% acetic acid and re-dehydrated at room temperature. The cells were lysed in a 10 mM Tris-buffered environment, and the OD was measured at a wavelength of 510 nm.

### 4.5. Real-Time PCR

Total RNA was isolated utilizing the RNeasy Mini Kit (Qiagen, Venlo, The Netherlands) following the manufacturer’s instructions. cDNA was synthesized using the High-Capacity cDNA Reverse Transcription Kit (Thermo Fisher Scientific). The Ct values were monitored across a spectrum using the Applied Biosystems 7300 Real-Time PCR system, employing Taqman gene expression assays for SF3A1 (Hs01066327) and STX12 (Hs00295291) in duplicate. The mRNA expression of 18S rRNA served as the normalization reference for each sample.

### 4.6. Xenografts

Immunodeficient BALB/c nude mice, aged 6 to 8 weeks, were purchased from The Jackson Laboratory, Japan. Mice were housed under controlled conditions (20–25 °C, 30–60% humidity) with a 12:12 h light/dark cycle and provided ad libitum access to food and water. The dorsal lateral region of male BALB/c nude mice was subjected to a subcutaneous injection of HCT116 cells (2 × 10^6^ cells). Experimental units were randomly allocated to control and treatment groups, and the order of treatments and outcome measurements was balanced across groups to mini-mize potential confounders. After the tumor tissue reached 4–5 mm, SF3A1 siRNA or scramble siRNA was administered daily using the GENOMONE-Si transfection kit (Ishihara Sangyo, Co., Ltd., Osaka, Japan) through local injection into the transplanted tumor. Tumor diameter was measured daily before transfection. The tumor sizes were calculated from digital caliper raw data using the following formula: Volume = (major tumor diameter) × (minor tumor diameter) × (minor tumor diameter)/2. Mice were euthanized by cervical dislocation following anesthesia with 5% isoflurane for induction. Animals were monitored at least once daily for clinical signs of distress, including body weight loss exceeding 20%, reduced activity, abnormal posture, and changes in general appearance. No animals met the predefined humane endpoint criteria, and no animals required early euthanasia.

### 4.7. Immunocytochemistry

HCT116 cells were cultured on glass chamber slides and subsequently fixed using a 4% paraformaldehyde solution at 4 °C. The cells underwent washing with PBS, followed by permeabilization using a 0.1% Triton X-100 solution and subsequent blocking with 3% BSA in PBS. Subsequently, the slides were incubated overnight at 4 °C with primary antibodies (Ki-67 [Novus, NB500-170]), followed by PBS washing and subsequent incubation with Alexa 488-conjugated secondary antibodies (Thermo Fisher Scientific) for 1 h at room temperature. The nuclei were counterstained with 4′,6-Diamidine-2′-phenylindole dihydrochloride (Sigma Aldrich).

### 4.8. TUNEL Staining

HCT116 cells were cultured on glass chamber slides and subsequently fixed with a 4% paraformaldehyde solution at 4 °C. The cells underwent PBS washing, followed by staining using an In Situ Cell Death Detection Kit with TMR red (Roche Diagnostic, Basel, Switzerland) according to the manufacturer’s instructions. The cells were mounted with an anti-fade mounting medium, and immunofluorescence was visualized using a fluorescence microscope (KEYENCE Corporation, Osaka, Japan).

### 4.9. RNA-Immunoprecipitation

HCT116 cells were solubilized in NP-40 cell lysis buffer (Thermo Fisher Scientific), supplemented with cOmplete™ Protease Inhibitor Cocktail (Merck, Darmstadt, Germany) and RNasin (Promega Corporation, Madison, WI, USA). The lysates were centrifuged at 20,000× *g* for 5 min to remove cellular debris. RNAs forming a complex with SF3A1 were selectively pulled down using either an SF3A1 antibody (Thermo Fisher Scientific; A301-602A) or an isotype control antibody, employing a Dynabeads immunoprecipitation kit (VERITAS Corporation, Santa Clara, CA, USA). The precipitated RNAs were isolated using phenol-chloroform extraction and purified with a mirVana™ Isolation Kit (Thermo Fisher Scientific).

### 4.10. Transcriptome Analyses

RNA libraries were generated using the Ion Total RNA-Seq Kit v2 (Thermo Fisher Scientific) following the manufacturer’s instructions. For RNA-seq, ribosomal RNA was depleted using the RiboMinus™ Eukaryote Kit v2 (Thermo Fisher Scientific) before library construction. The RNA libraries underwent an emulsion polymerase chain reaction (PCR) process utilizing the Ion OneTouchTM system and the Ion OneTouch 200 Template kit v3 (Thermo Fisher Scientific). Template-positive Ion SphereTM particles were enriched and purified, prepared for subsequent sequencing using the Ion OneTouchTM ES system (Thermo Fisher Scientific). Following this, the template-positive Ion SphereTM Particles were loaded onto Ion PITM Chips (Thermo Fisher Scientific), and high-throughput sequencing was performed using the Ion Proton™ Semiconductor sequencer (Thermo Fisher Scientific). The entire sequencing dataset was aligned to the human reference genome sequence (GRCh37/hg19) using the Torrent Suite software program version 5.18 (Thermo Fisher Scientific). Gene-level expression values for each sample were imported into CLC Genomics Workbench (CLC bio, Aarhus, Denmark) for downstream analysis. Differential expression was assessed using unpaired *t*-tests, and significance thresholds were defined as an adjusted *p*-value < 0.01. To integrate the RIP-seq and RNA-seq datasets, transcripts with >2-fold enrichment in RIP-seq and significantly changed expression (absolute fold change > 2) upon SF3A1 knockdown in RNA-seq were considered potential direct targets.

### 4.11. Western Blotting

HCT116 cells were lysed in NP-40 cell lysis buffer (Thermo Fisher Scientific) supplemented with cOmplete™ Protease Inhibitor Cocktail (Merck). Following centrifugation at 20,000× *g* for 5 min, the lysate was denatured with Laemmli Sample Buffer containing 2-mercaptoethanol at 95 °C for 5 min. Equal amounts of protein were loaded onto an SDS–PAGE gel (12.5%), followed by transfer onto a nitrocellulose membrane at 100 V for 60 min. The blots were blocked in SuperBlock T-20 (PBS; Thermo Fisher Scientific) for 1 h, then incubated with the primary antibody in SuperBlock T-20. The primary antibody used, specifically the cleaved PARP (#9546, Cell Signaling Technology, Inc., Danvers, MA, USA), was diluted to 1:1000 in SuperBlock T-20 (PBS). Subsequently, an overnight incubation with the blots was conducted at 4 °C. The blots were washed in 0.05% Tween20-PBS (T-PBS) three times for 15 min and then incubated in SuperBlock T-20 (PBS) containing HRP-conjugated secondary antibodies (R&D Systems, Inc., Minneapolis, MN, USA). Following three washes in T-PBS for 15 min each, the blots were visualized using the Super Signal West Pico enhanced chemiluminescence system (Thermo Fisher Scientific). Actin (612656, BD Transduction Laboratories, Franklin Lakes, NJ, USA) protein expression was used for normalizing protein levels. Uncropped images of each blot are shown in Figure S11.

### 4.12. Caspase-Glo 3/7 Assay

Cells were treated with glucose oxidase, and caspase activity was measured using a Caspase-Glo 3/7 assay kit (Promega, Madison, WI, USA) according to the manufacturer’s instructions.

### 4.13. Luciferase Reporter Assay

The full-length STX12 mRNA (NCBI Reference Sequence: NM_177424.3, nucleotides 1–2893) was cloned into the multiple cloning sites SacI and XhoI of the pmirGLO vector (Promega). A total of 2 × 10^5^ HCT116 cells were seeded in 6-well plates and transfected 24 h later with either the STX12 reporter construct or an empty vector using Lipofectamine 3000 (Thermo Fisher Scientific). After an additional 24 h, cells were harvested by trypsinization, and 1 × 10^4^ cells were reseeded and reverse-transfected with either SF3A1 siRNA or scrambled control RNA using Lipofectamine RNAiMAX (Thermo Fisher Scientific), following the manufacturer’s reverse transfection protocol. Twenty-four hours post-transfection, cells were subjected to the Luciferase Assay System (Promega), and luminescence was measured using an Enspire microplate reader.

### 4.14. Statistical Analyses

All data are presented as mean ± standard deviation (SD). Statistical analyses were performed using Student’s *t*-test or one-way analysis of variance (ANOVA), followed by Dunnett’s or Tukey’s post hoc test where appropriate. A *p*-value < 0.05 was considered statistically significant. Data analysis was performed using GraphPad Prism version 10.4.2.

## 5. Conclusions

Our findings demonstrate that SF3A1 contributes to CRC progression through stabilization of STX12 mRNA, thereby inhibiting apoptosis selectively in CRC cells. Importantly, although SF3A1 is expressed at similar levels in cancerous and non-cancerous tissues, its functional role is dramatically altered in tumor cells, likely due to PTMs. Our study identifies the SF3A1–STX12 regulatory axis as a novel and selective therapeutic target for colorectal cancer.

## Figures and Tables

**Table 1:** Gene ontology (GO) analysis of 307 downregulated mRNA.

Category	Term	*p*-Value
GOTERM_BP_DIRECT	GO:1901255~nucleotide-excision repair involved in interstrand cross-link repair	0.018047672
GOTERM_BP_DIRECT	GO:0010613~positive regulation of cardiac muscle hypertrophy	0.018243629
GOTERM_BP_DIRECT	GO:0050729~positive regulation of inflammatory response	0.018459991
GOTERM_BP_DIRECT	GO:0051216~cartilage development	0.02121953
GOTERM_BP_DIRECT	GO:0090263~positive regulation of canonical Wnt signaling pathway	0.023163347
GOTERM_BP_DIRECT	GO:0051092~positive regulation of NF-kappaB transcription factor activity	0.025098147
GOTERM_CC_DIRECT	GO:0000110~nucleotide-excision repair factor 1 complex	0.026617289
GOTERM_BP_DIRECT	GO:0010041~response to iron(III) ion	0.026949681
GOTERM_MF_DIRECT	GO:0003684~damaged DNA binding	0.02964456
GOTERM_CC_DIRECT	GO:0055037~recycling endosome	0.034071256
GOTERM_CC_DIRECT	GO:0016323~basolateral plasma membrane	0.035217237
GOTERM_BP_DIRECT	GO:0006537~glutamate biosynthetic process	0.035771443
GOTERM_BP_DIRECT	GO:0046452~dihydrofolate metabolic process	0.035771443
GOTERM_CC_DIRECT	GO:0031901~early endosome membrane	0.038822366
GOTERM_CC_DIRECT	GO:0030670~phagocytic vesicle membrane	0.042076539
GOTERM_BP_DIRECT	GO:0045595~regulation of cell differentiation	0.042088297
GOTERM_CC_DIRECT	GO:0097180~serine protease inhibitor complex	0.043969464
GOTERM_MF_DIRECT	GO:0046872~metal ion binding	0.047743271

**Table 2:** The top 10 mRNAs with the largest changes.

	IP-Transcriptome Analysis (SF3A1/IgG)		Transcriptome Analysis (SF3A1/Scramble)	
	Fold Change	*p*-Value	Fold Change	*p*-Value
MGC32805	30.48	0.0008	−9.19	0.013
DRICH1	------	0.0246	−8.30	0.011
DNAJC5G	7.15	0.0036	−6.74	0.008
SLCO1B3	61.46	0.0137	−5.62	0.012
GEMIN2	3.35	0.0157	−5.51	0.005
EIF4EBP3	6.29	0.0228	−5.50	0.003
MRPS31P	21.88	0.0090	−5.00	0.006
STX12	4.63	0.0069	−4.90	0.007
VIM-AS1	7.42	0.0139	−4.90	0.023
LUCAT1	24.73	0.0011	−4.59	0.012

## Data Availability

Raw data were generated at the Division of Gastroenterology, Department of Internal Medicine, Asahikawa Medical University. Derived data supporting the findings of this study are available from the corresponding author, H.K., on request. The datasets generated and/or analyzed during the current study are available in the Gene Expression Omnibus (GEO), GSE282463.

## Supplementary Materials

The following supporting information can be downloaded at: https://www.mdpi.com/article/10.3390/ijms27031195/s1.

## Abbreviations

The following abbreviations are used in this manuscript:

5-FU	5-fluorouracil
ANOVA	analysis of variance
CRC	colorectal cancer
FBS	fetal bovine serum
GO	Gene ontology
OD	optical density
PARP	poly ADP-ribose polymerase
PCR	polymerase chain reaction
PI3K	phosphatidylinositol 3-kinase
PK	pyruvate kinase
PTB	polypyrimidine tract-binding protein
PTM	Post-translational modifications
RBPs	RNA-binding proteins
RIP	RNA-immunoprecipitation
SD	standard deviation
SF3A1	Splicing factor 3A1
SRB	sulforhodamine B
STX12	Syntaxin12
TCA	trichloroacetic acid
TUNEL	TdT dUTP-mediated nick-end labeling
hnRNP	heterogeneous nuclear ribonucleoprotein
snRNP	small nuclear ribonucleoprotein
