# Supplementary figures and images for: Splicing Factor 3a Subunit 1 Promotes Colorectal Cancer Growth via Anti-Apoptotic Effects of Syntaxin12
Source: Int J Mol Sci. 2026 Jan 24;27(3):1195. doi: 10.3390/ijms27031195 (PMC12897669; doi:10.3390/ijms27031195)

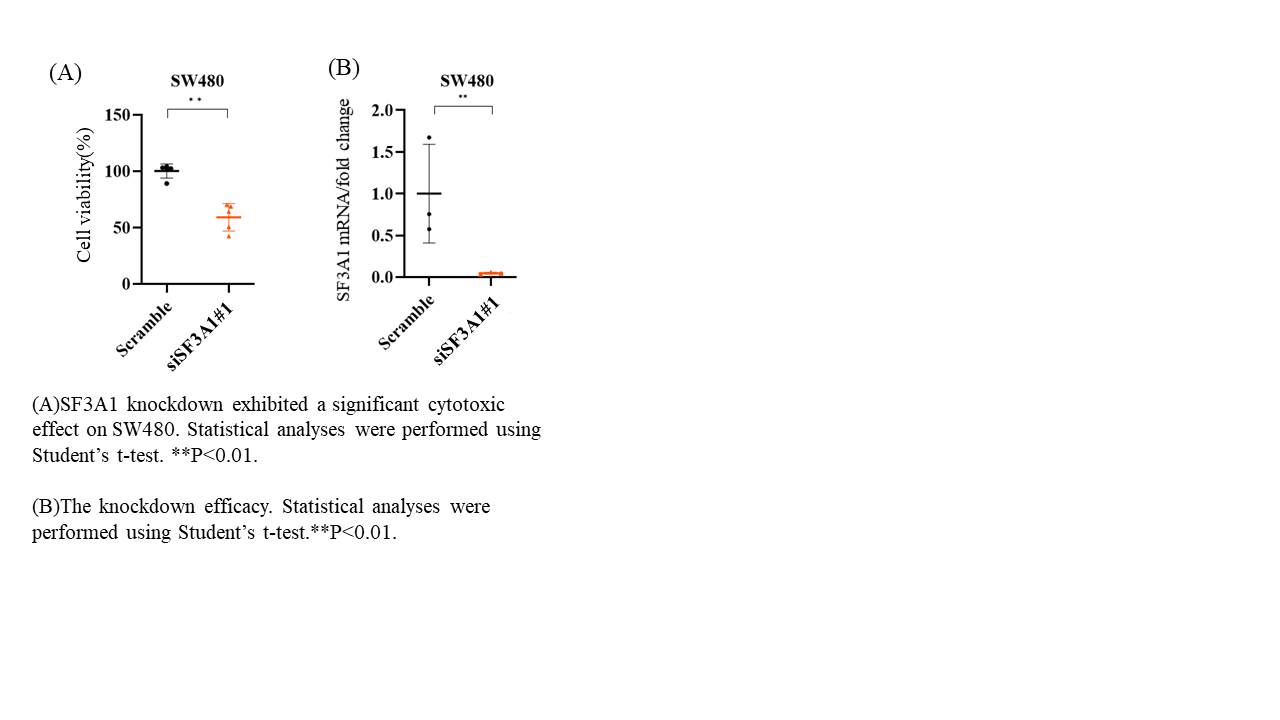

Supplement: Supplementary file 1 [file ijms-27-01195-s001.zip › Supplemental figure S1.TIF]

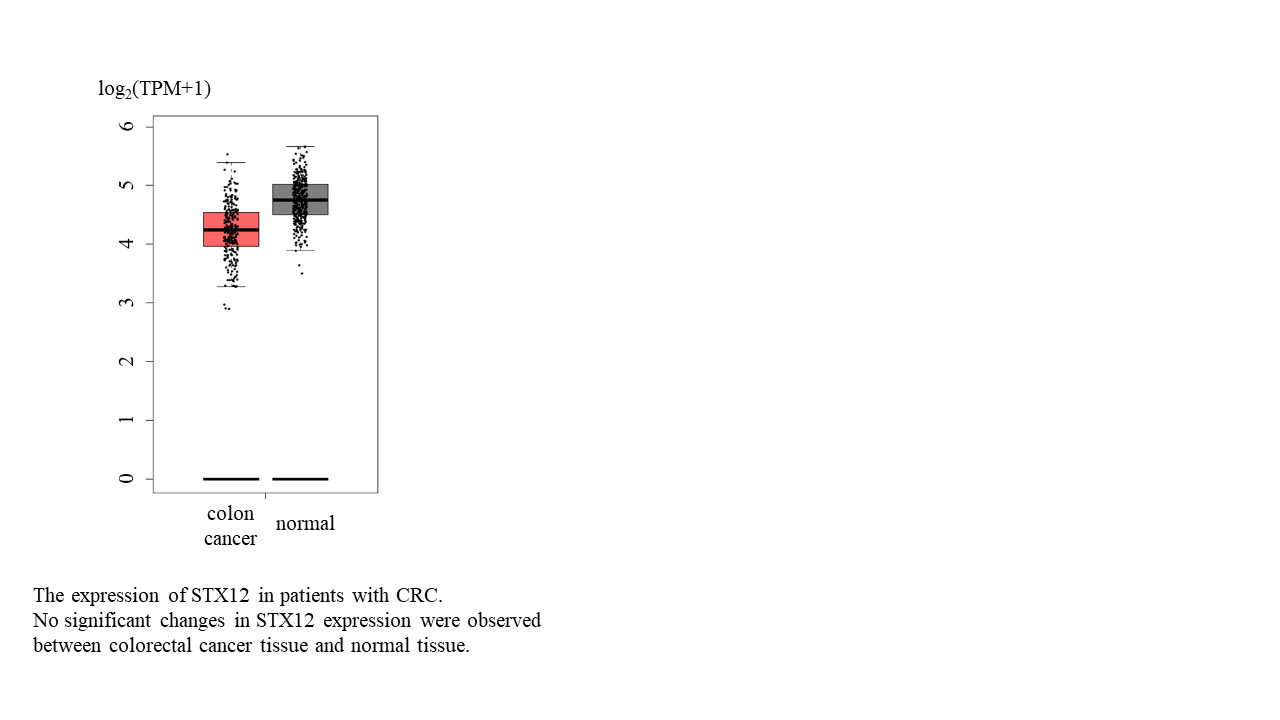

Supplement: Supplementary file 1 [file ijms-27-01195-s001.zip › Supplemental figure S10.TIF]

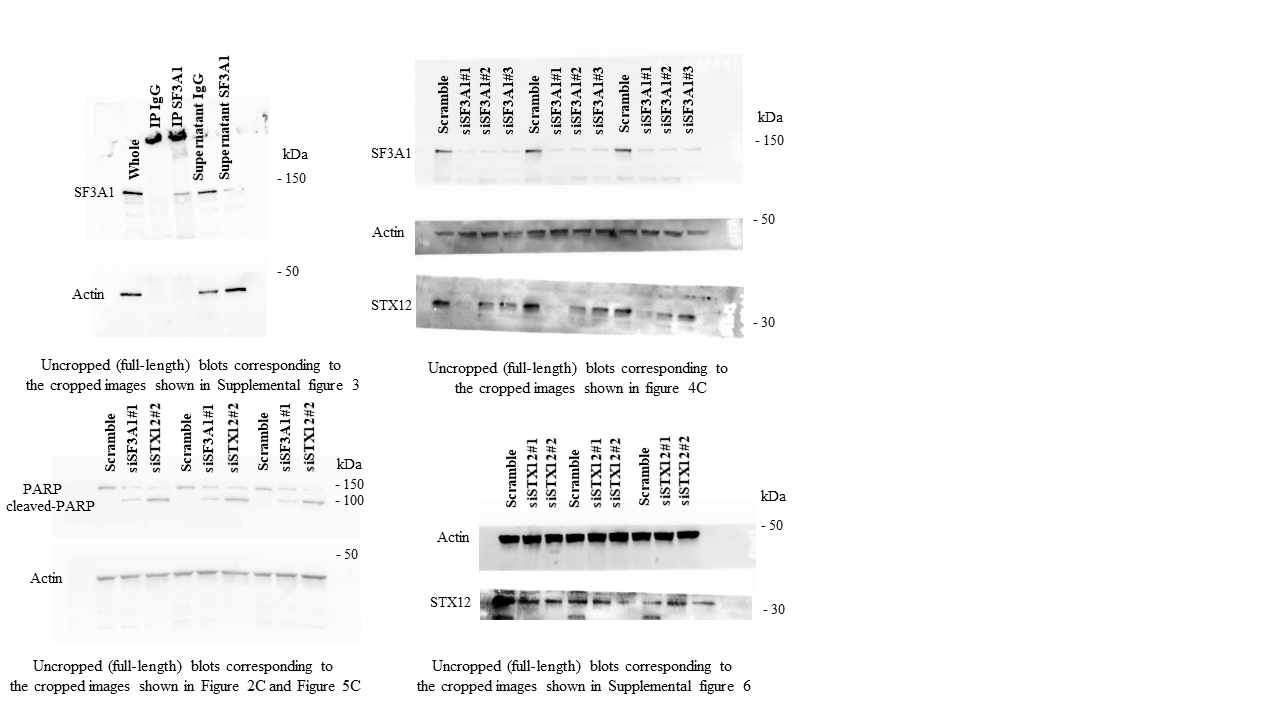

Supplement: Supplementary file 1 [file ijms-27-01195-s001.zip › Supplemental figure S11.TIF]

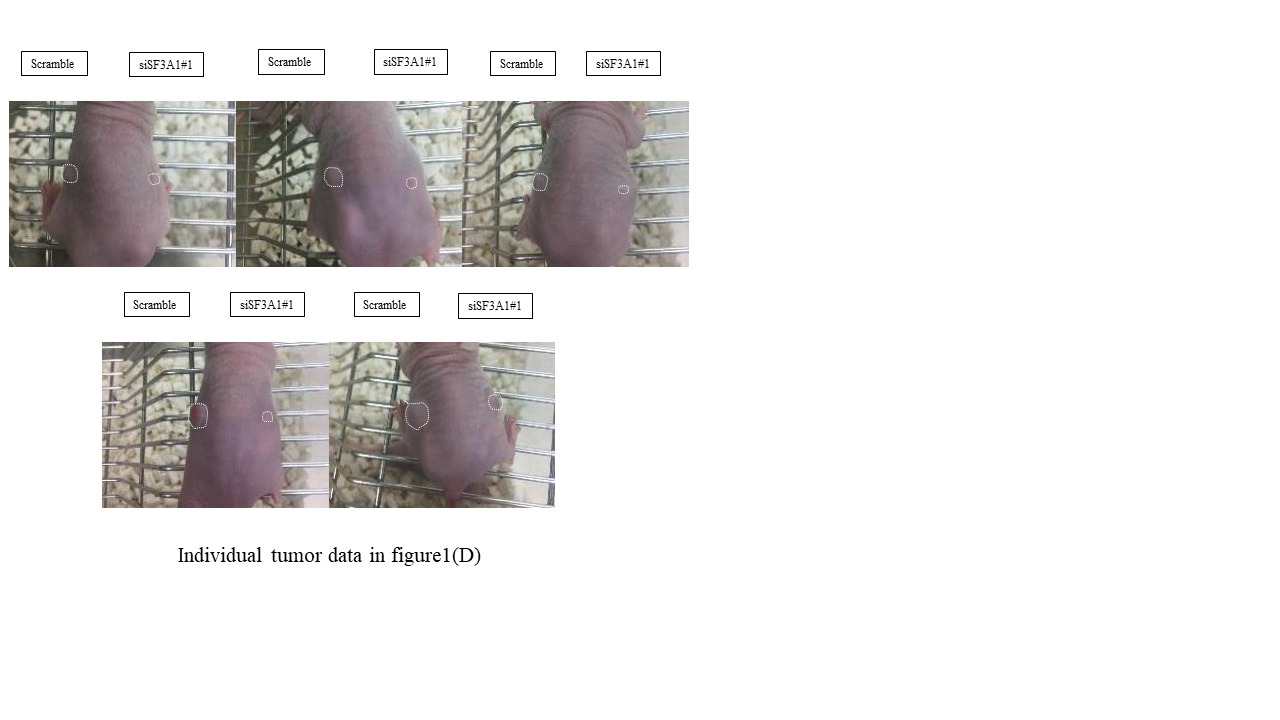

Supplement: Supplementary file 1 [file ijms-27-01195-s001.zip › Supplemental figure S2.TIF]

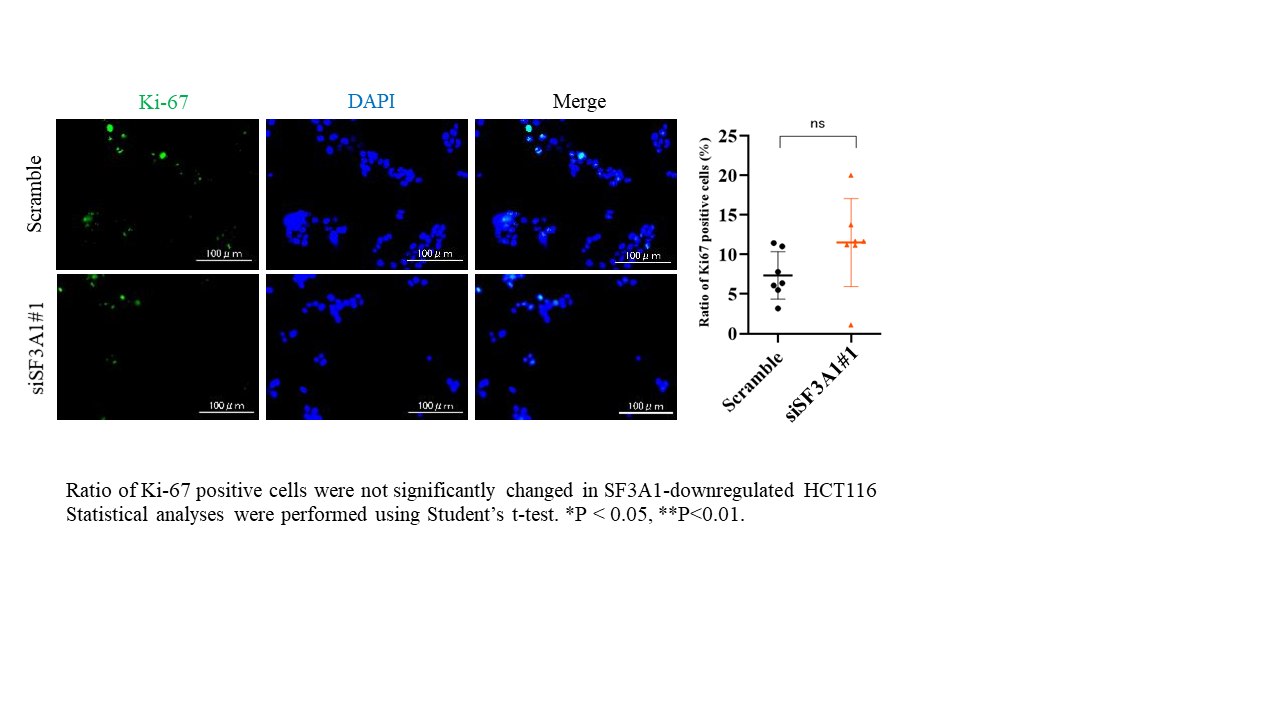

Supplement: Supplementary file 1 [file ijms-27-01195-s001.zip › Supplemental figure S3.TIF]

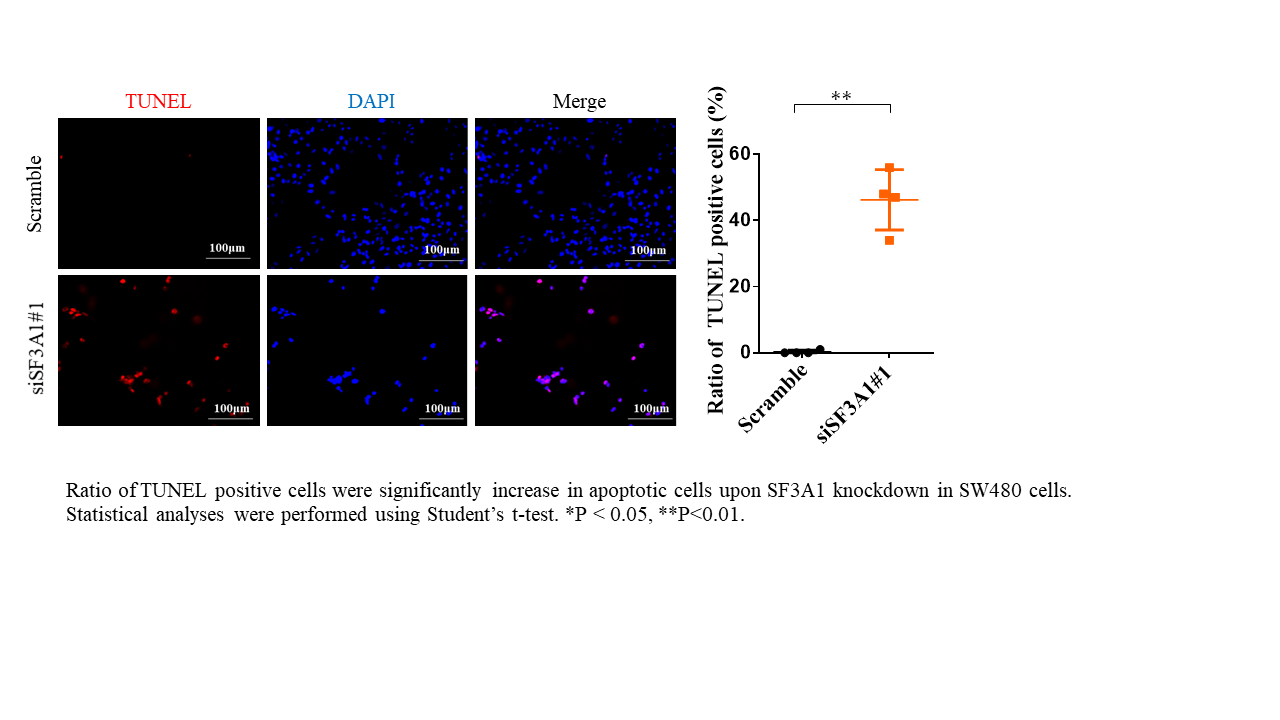

Supplement: Supplementary file 1 [file ijms-27-01195-s001.zip › Supplemental figure S4.tif]

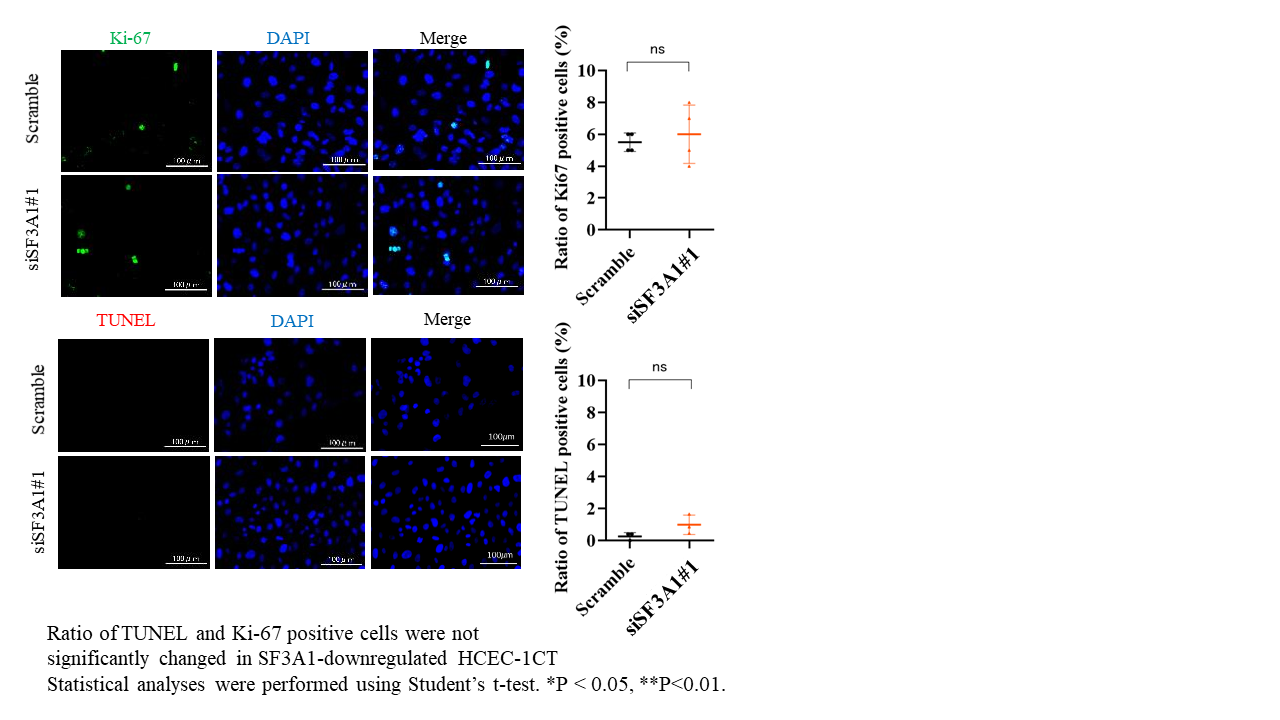

Supplement: Supplementary file 1 [file ijms-27-01195-s001.zip › Supplemental figure S5.TIF]

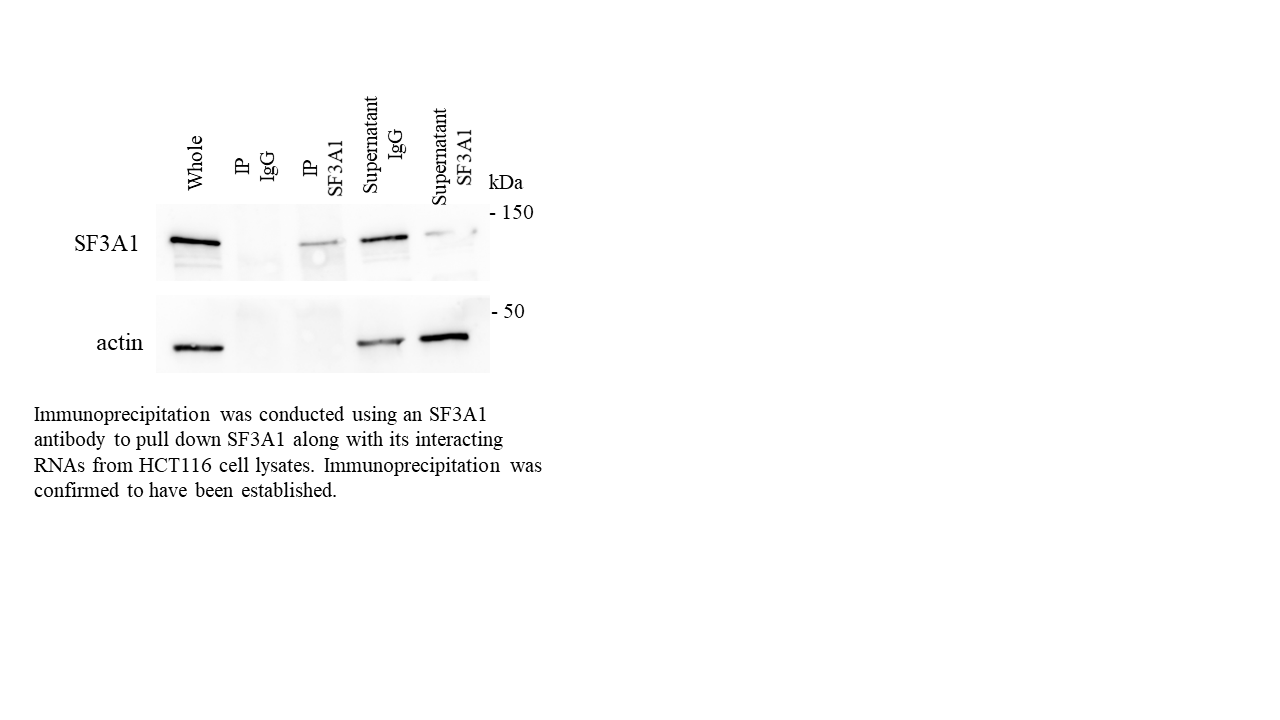

Supplement: Supplementary file 1 [file ijms-27-01195-s001.zip › Supplemental figure S6.TIF]

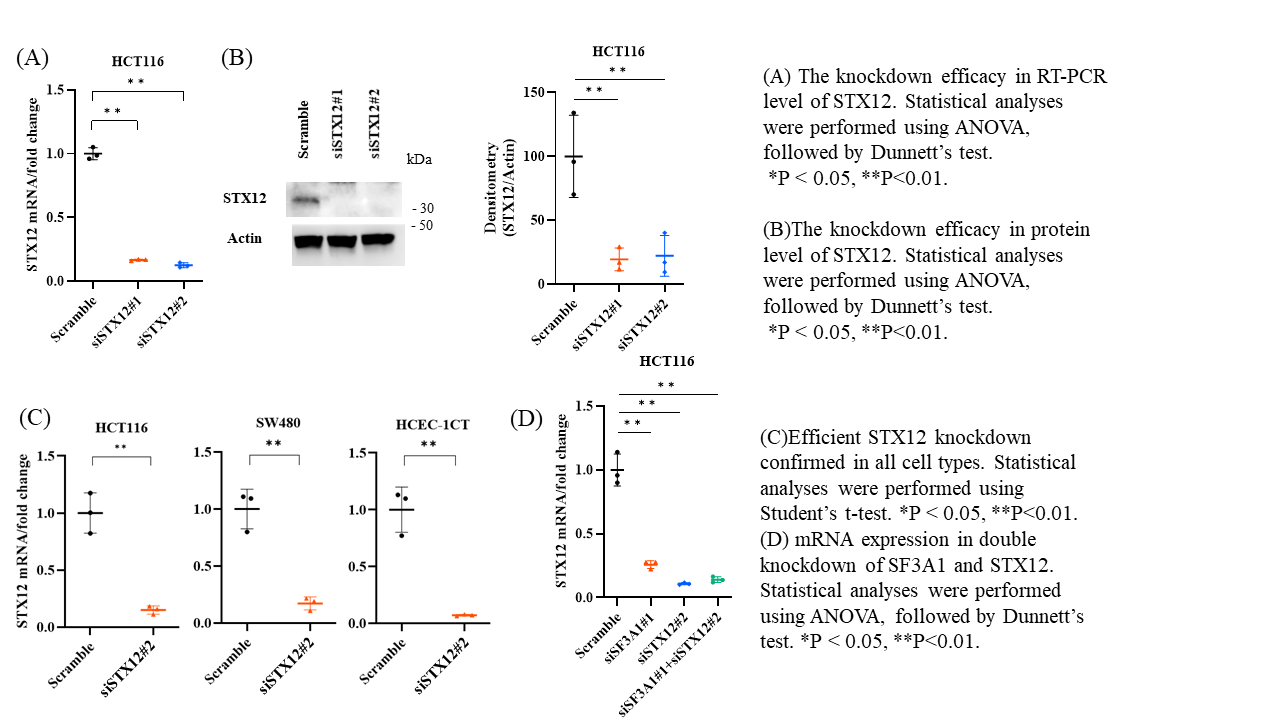

Supplement: Supplementary file 1 [file ijms-27-01195-s001.zip › Supplemental figure S7.TIF]

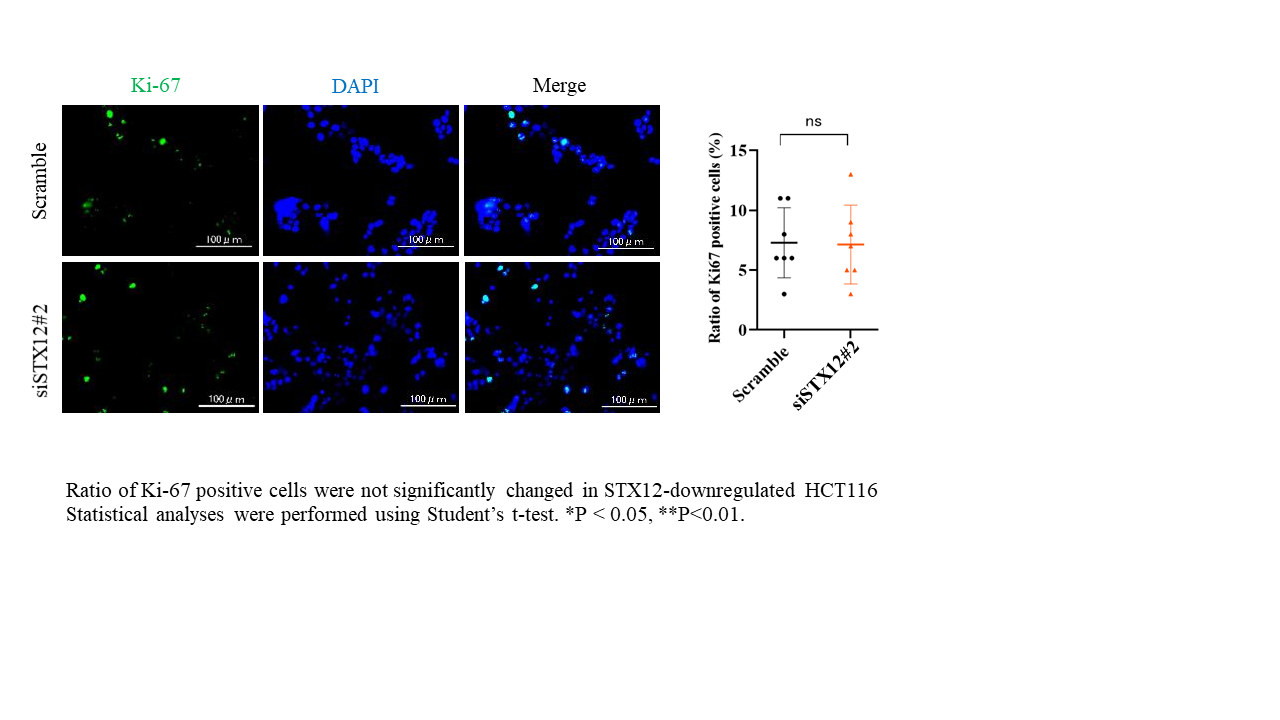

Supplement: Supplementary file 1 [file ijms-27-01195-s001.zip › Supplemental figure S8.TIF]

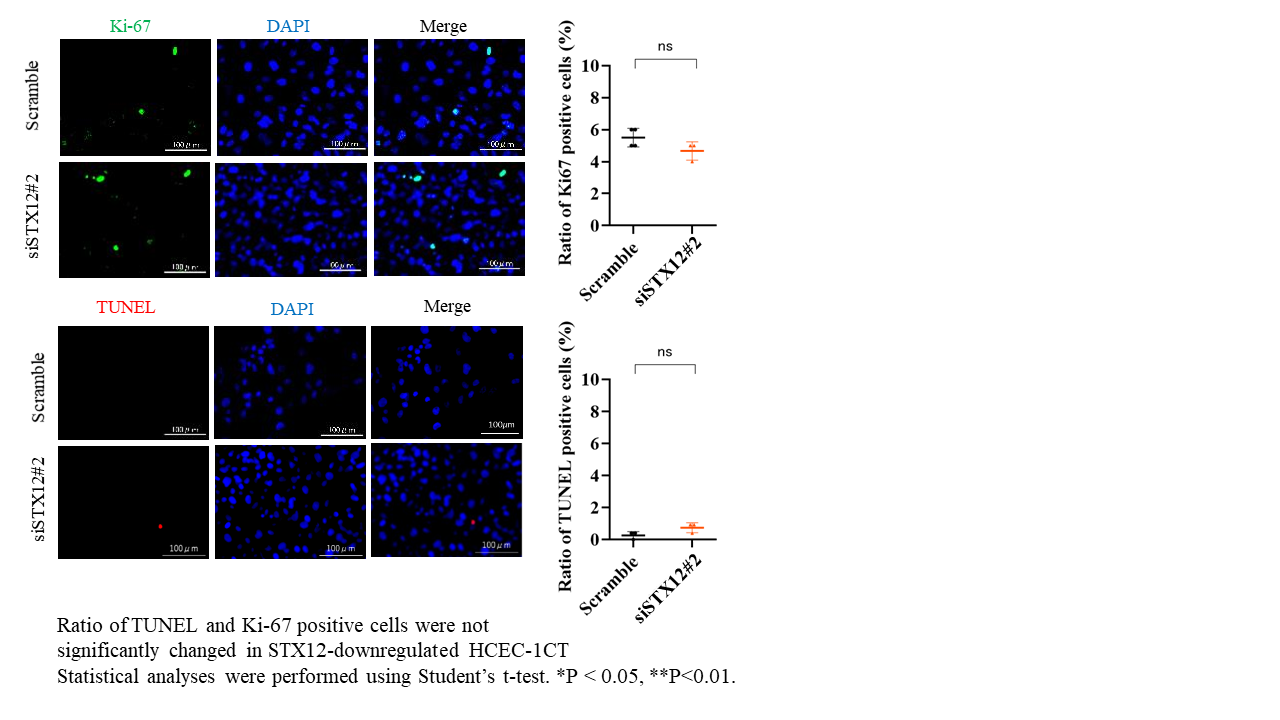

Supplement: Supplementary file 1 [file ijms-27-01195-s001.zip › Supplemental figure S9.TIF]
